# Diagnostic value of CA125, HE4, and systemic immune-inflammation index in the preoperative investigation of ovarian masses

**DOI:** 10.1097/MD.0000000000035240

**Published:** 2023-09-15

**Authors:** Liyun Song, Jie Qi, Jing Zhao, Suning Bai, Qi Wu, Ren Xu

**Affiliations:** a Department of Gynecology, Hebei General Hospital, Shijiazhuang, China.

**Keywords:** CA125, diagnosis, fibrinogen-to-albumin ratio, HE4, ovarian cancer, prognostic nutritional index, systemic immune-inflammation index

## Abstract

This study aimed to ascertain the diagnostic accuracy of CA125, HE4, systemic immune-inflammation index (SII), fibrinogen-to-albumin ratio (FAR), prognostic nutritional index (PNI), and their combination for ovarian cancer (OC) to discover an optimal combined diagnostic index for early diagnosis of OC. A thorough investigation was conducted to ascertain the correlation between these markers and the pathological characteristics of OC, thereby providing a foundation for early identification and treatment of this disorder. One hundred seventy patients with documented OC and benign ovarian tumors (BOTs) treated at Hebei General Hospital between January 2019 and December 2022 were included in this retrospective study. Data analysis was conducted using IBM SPSS Statistics version V26.0, MedCalc Statistical Software version 19.4.0, and the R Environment for Statistical Computing software (R Foundation for Statistical Computing). Isolated CA125 showed the best application value for differentiating benign ovarian tumors from OC when the defined variables were compared separately. The combination of CA125, HE4, FAR, SII, and PNI displayed a greater area under the operating characteristic curve curve than any one of them or other combinations of the 5 variables. Compared to CA125 alone, the combination of CA125, HE4, FAR, SII, and PNI showed a slight gain in sensitivity (83.91%), negative predictive value (83.91%), accuracy (85.88%), and a decrease in negative likelihood ratio (0.180%). Higher preoperative CA125, HE4, SII, and FAR levels, and lower PNI levels predicted a higher probability of advanced OC progression and lymph node metastasis. FAR has better application value than other inflammation-related markers (PNI and SII). This study suggests that preoperative serum SII, PNI, and FAR may be clinically valuable markers in patients with OC. FAR has better application value than other inflammation-related markers (PNI and SII). As we delve deeper into the inflammatory mechanisms associated with tumors, we may discover more effective combinations of tumor and inflammatory biomarkers.

## 1. Introduction

Ovarian cancer (OC) is the fifth leading cause of cancer-related death in females.^[1]^ In recent years, OC has been on the rise.^[2]^ The prognosis of OC is poor, with a survival rate of only 30%.^[3]^ As the early manifestations of OC are rather hidden, the disease may have developed to a middle or advanced stage at diagnosis, resulting in missed optimal treatment timing and increased mortality.^[4–6]^ The 5-year survival rate of OC can be as high as 90% when detected early and treated with standard surgery and adjuvant therapy.^[7]^ Differentiating malignant from benign ovarian tumors (BOTs) is imperative.

Efforts have been made to identify more reliable biomarkers for the early detection of OC, and serum biomarkers are a practical, cost-effective, and noninvasive approach for predicting malignancy. CA125 is expressed in over 80% of OC patients and can be detected in the serum, thus enabling the differentiation of malignant ovarian tumors from normal ovarian tissues.^[8]^ However, this marker has low sensitivity in the early stages of OC.^[9]^ In addition, it has a high false-positive rate in benign gynecological conditions such as acute pelvic inflammation, adenomyosis, uterine myoma, and endometriosis.^[10]^ Additional biomarkers, such as HE4, have been developed to enhance ovarian carcinoma’s specificity.^[11]^ This biomarker is overexpressed in OC tissues.^[12]^ In addition to colorectal cancer and gastrointestinal malignancies, HE4 is a nonspecific tumor marker expressed to varying degrees in cervical, endometrial, ovarian, and non-epithelial tumors.^[13]^ It is strongly associated with tumor invasion, migration, and recurrence. The 2 most effective currently available markers, CA125 and HE4, are insufficient for detecting early-stage OC.^[14–16]^ A great deal of effort is being put forth to discover additional biomarkers that, either alone or in combination with CA125 and HE4, could enhance the sensitivity and specificity of OC detection in a timely and treatable manner.

The behavior of OC, in which the body’s inflammatory and immune responses play a crucial role, has also recently been better understood by the scientific community.^[17]^ The inflammatory response is significantly affected by the immunological and nutritional state of the body, and the presence and metastatic spread of tumor cells are closely linked to inflammation. Malnutrition has been reported to make patients more susceptible to infection and to promote tumor recurrence through the suppression of tumor immunity.^[18,19]^ Therefore, a growing number of studies have focused on how nutrition and inflammation interact in cancer patients. Studies have shown that peripheral blood neutrophils, lymphocytes, platelets, albumin, globulin, and fibrinogen play essential roles in the inflammatory microenvironment of cancer.^[20]^ Recently, the preoperative systemic immune-inflammation index (SII), prognostic nutritional index (PNI), and fibrinogen-to-albumin ratio (FAR) have been identified as significant indicators of the diagnostic utility and prognosis of prostate cancer, lung cancer, and gastrointestinal tumors.^[21–23]^ However, the role of inflammation-related indicators in the diagnosis of ovarian cancer has rarely been reported. To the best of our knowledge, no study has been published up to now that examined the clinical utility of CA125 combined with HE4, SII, PNI, and FAR in predicting OC in the preoperative setting. This study aimed to ascertain the diagnostic accuracy of CA125, HE4, SII, FAR, PNI, and their combinations for OC to discover an optimal combined diagnostic index for the early diagnosis of OC. A thorough investigation was conducted to ascertain the correlation between these markers and the pathological characteristics of OC, thereby providing a foundation for early identification and treatment of this disorder.

## 2. Materials and methods

### 2.1. Inclusion and exclusion criteria

This retrospective study included 170 patients with documented OC and BOTs who were treated at Hebei General Hospital between January 2019 and December 2022, and were divided into 2 groups of 87 OC and 83 BOTs based on postoperative pathological results reviewed by 2 senior pathologists. The patients with OC did not receive chemotherapy or radiation therapy before surgery. The International Federation of Gynecology and Obstetrics staging system was used to determine the clinical stage of OC. All enrolled patients underwent comprehensive staging surgery including, total hysterectomy, adnexectomy, complete pelvic/para-aortic lymphadenectomy, and peritoneal cytology. Patients with infectious conditions, autoimmune diseases, severe liver or kidney damage, thrombotic diseases, other benign or malignant tumors, pregnancy, or preoperative complications of blood diseases were excluded from the study.

### 2.2. Clinical and laboratory data collection

The data analyzed consisted of clinical and laboratory factors such as age, pathological type, International Federation of Gynecology and Obstetrics staging, degree of tissue differentiation, presence or absence of lymph node metastasis, albumin, fibrinogen, neutrophil count, platelet count, lymphocyte count, CA125, and HE4. Prior to surgery, albumin, fibrinogen, neutrophil count, platelet count, lymphocyte count, and serum tumor biomarkers were all tested and recorded within a week. Using a COBAS E602 analyzer (Roche, Switzerland) and a chemiluminescent reagent kit (Roche, Switzerland), preoperative CA125 and HE4 concentrations were measured. Two senior pathologists reviewed the pathological examinations, and the Ethics Committee of Hebei General Hospital approved the collection of patients clinical and laboratory data, following the Declaration of Helsinki. The ethical committee’s conclusion that informed consent was not necessarily meant that written informed consent was no longer required.

### 2.3. Inflammation-related markers

The formula for serum inflammation-related markers was: FAR = fibrinogen(g/L)/albumin(g/L), PNI = albumin (g/L) + 5 × lymphocyte count (10^9^/L), and SII = platelet count (10^9^/L) × neutrophil count (10^9^/L)/ lymphocyte count (10^9^/L).

### 2.4. Statistical analysis

Data were analyzed using IBM SPSS statistics version V26.0, MedCalc Statistical Software version 19.4.0, and R Environment for Statistical Computing software (R Foundation for Statistical Computing). Statistical significance was set at *P* < .05, and *P* values were calculated. The Shapiro–Wilk test was used to assess the normality of the distribution of variables. The data are presented as the mean ± SD for continuous variables with normal distribution and as the median and interquartile range for continuous variables without normal distribution. To assess the differences in variables among the groups, the Kruskal–Wallis test was used, followed by evaluation using the Mann–Whitney *U* test for multiple comparisons. The area under the operating characteristic curve (ROC), 95% confidence interval, sensitivity, specificity, positive predictive value (PPV), negative predictive value (NPV), positive likelihood ratio (LR), negative LR, and accuracy for the defined variables were calculated to test the diagnostic performance for OC prediction by receiver ROC analysis. The Youden index of the ROC curve was then used to determine the optimal cutoff value of the parameters. Spearman rank correlation test and logistic regression analysis were used to assess the associations between pertinent parameters.

Subsequently, decision curve analysis (DCA) was conducted to determine which single parameter and parameter combinations provided the most clinical utility in distinguishing BOTs from OC. For this visual analysis, we used software explicitly designed for DCA. All DCA calculations were performed as described by Vickers and Elkin.^[24]^

## 3. Results

### 3.1. CA125, HE4, SII, PNI, and FAR showed significant differences among the benign ovarian tumor group and OC group

In this study, 170 patients with ovarian tumors were enrolled, and the primary laboratory parameters of all participants are summarized in Table 1. The overall malignancy prevalence of our cohort was 51.18%, with the OC group having a mean age of 52.22 ± 11.26 years (range, 21–76 years) and the control group having a mean age of 51.71 ± 12.20 years (range, 26–78 years). There were no significant differences between the 2 groups with respect to age (t = −0.282, *P* = .778). Table 1 reveals a significant difference between patients with BOTs and OC in terms of absolute neutrophil count, absolute lymphocyte count, blood platelet count, albumin, fibrinogen, SII, PNI, FAR, CA125, and HE4 (*P* = .001, *P* = .001, *P* = .001, *P =* .001, *P* < .001, *P* < .001, *P* < .001, *P* < .001, *P* < .001, *P* < .001, respectively). The median CA125, HE4, SII, and FAR values were found to be significantly higher in the OC group [884.86 (859.31), 0.087 (0.049), 228.50 (777.87), 180.20 (308.80)] compared to those in the BOT group [567.82 (354.31), 0.062 (0.017), 16.43 (13.85), 45.90 (17.22)]. Conversely, the median PNI value was significantly lower in the OC group [46.40 (8.10)] than in the BOT group [51.20 (5.10)].

**Table 1:** Comparison of defined variables between ovarian cancer and benign ovarian tumor.

Variables	Ovarian cancer, median (IQR)	Benign tumor, median (IQR)	Reference level	Z value	*P* value
Number	87	83			
N (109/L)	4.20 (1.97)	3.45 (2.02)	1.8–6.3	−3.361	.001
L (109/L)	1.41 (0.68)	1.72 (0.68)	1.1–3.2	−3.378	.001
PLT (109/L)	311.00 (125.00)	258.00 (92.00)	125–350	−3.248	.001
Alb(g/L)	39.70 (5.97)	42.60 (4.60)	40–55	−3.451	.001
Fib(g/L)	3.59 (1.54)	2.73 (0.85)	2–4	−6.50	<.001
SII	884.86 (859.31)	567.82 (354.31)	/	−5.379	<.001
PNI	46.40 (8.10)	51.20 (5.10)	/	−4.322	<.001
FAR	0.087 (0.049)	0.062 (0.017)	/	−6.586	<.001
CA125 (U/mL)	228.50 (777.87)	16.43 (13.85)	0–35	−8.780	<.001
HE4 (pmol/L)	180.20 (308.80)	45.90 (17.22)	Premenopause < 70 Postmenopause < 140	−8.080	<.001

Alb = albumin, FAR = fibrinogen(g/L)/ albumin(g/L), Fib = fibrinogen, IQR = interquartile range, L = absolute lymphocyte count, N = absolute neutrophil count, PLT = blood platelet count, PNI = albumin (g/L) + 5 × lymphocyte count (10^9^/L), SII = platelet count (10^9^/L) × neutrophil count (10^9^/L)/ lymphocyte count (10^9^/L).

### 3.2. Correlation between CA125, HE4, SII, PNI, FAR, and clinic-pathological characteristics of OC patients

Table 2 displays the histopathology and characteristics of the OC-enrolled patients with differentiation grades and cancer stages of OC. A comparison of CA125, HE4, SII, PNI, FAR, and clinical characteristics between the BOT and OC groups is shown in Table 2. Compared with the early-stage OC (Stage I–II) group, the values of CA125 [107.09 (267.10) vs 618.40 (1086.20), (Stage I–II) vs (Stage III–IV), *P<*.001], HE4 [82.80 (153.37) vs 299.00 (816.95), (Stage I–II) vs (Stage III–IV), *P<*.001], SII [685.64 (511.86) vs 1252.37 (857.18), (Stage I–II) vs (Stage III–IV), *P<*.001], and FAR [0.08 (0.04) vs 0.11 (0.06), (Stage I–II) vs (Stage III–IV), *P* = .001] in the advanced OC (Stage III–IV) group were significantly higher, while the value of PNI [49.90 (8.77) vs 45.05(8.08), (Stage I–II) vs (Stage III–IV), *P* = .001] in the advanced OC (Stage III–IV) group was significantly lower. Moreover, compared with the non-lymph node metastasis OC group, the values of CA125 [161.90 (345.05) vs 714.50 (1074.40), (non-lymph node metastasis) vs (lymph node metastasis), *P<*.001], HE4 [84.65 (165.88) vs 331.00 (859.80), (non-lymph node metastasis) vs (lymph node metastasis), *P<*.001], SII [711.42 (552.86) vs 1332.02 (999.87), (non-lymph node metastasis) vs (lymph node metastasis), *P<*.001], and FAR [0.08 (0.03) vs 0.12 (0.08),(non-lymph node metastasis) vs (lymph node metastasis), *P* = .001] in the lymph node metastasis OC group were significantly higher, while the value of PNI [49.45 (7.45) vs 44.45 (5.20), (Lymph nodes negative) vs (Lymph nodes positive), *P* = .002] was significantly lower. These results suggest that higher preoperative CA125, HE4, SII, and FAR levels, and lower PNI levels predict a higher probability of advanced OC progression and lymph node metastasis.

**Table 2:** Relationship between laboratory variables and clinic-pathological characteristics of OC patients.

Variables	N (%)	CA125 (U/mL), median (IQR)	HE4 (pmol/L), median (IQR)	SII, median (IQR)	PNI, median (IQR)	FAR, median (IQR)
Age						
≤50	30 (34.48%)	329.90 (878.63)	174.30 (317.61)	795.75 (797.02)	49.13 (6.85)	0.08 (0.04)
> 50	57 (65.52%)	215.80 (683.83)	180.20 (329.31)	947.76 (938.70)	45.85 (9.42)	0.09 (0.06)
Z value		−0.040	−0.621	−0.406	−1.072	−0.380
*P* value		.968	.535	.685	.284	.704
FIGO staging						
I–II	42 (48.28%)	107.09 (267.10)	82.80 (153.37)	685.64 (511.86)	49.90 (8.77)	0.08 (0.04)
III–IV	45 (51.72%)	618.40 (1086.20)	299.00 (816.95)	1252.37 (857.18)	45.05 (8.08)	0.11 (0.06)
Z value		−4.672	−4.196	−3.610	−3.462	−3.381
*P* value		*<*.001	*<*.001	*<*.001	.001	.001
Histological grade						
G1	29 (33.33%)	121.15 (490.74)	66.99 (151.25)	829.65 (779.16)	46.23 (12.94)	0.09 (0.07)
G2–G3	58 (66.67%)	307.00 (784.60)	206.90 (339.60)	947.76 (931.14)	46.90 (6.52)	0.09 (0.05)
Z value		−1.972	−3.134	−1.054	−0.232	−0.790
*P* value		.049	.002	.292	.817	.429
Pathological type						
Serous	68 (78.15%)	294.40 (777.60)	204.65 (363.93)	962.18 (928.09)	46.08 (7.18)	0.09 (0.05)
Mucinous	8 (9.20%)	32.21 (177.89)	52.43 (39.66)	704.91 (729.60)	54.00 (14.78)	0.08 (0.06)
Clear-cell	5 (5.75%)	29.28 (342.21)	39.10 (82.82)	676.47 (652.84)	49.70 (16.15)	0.09 (0.08)
Others	6 (6.90%)	675.20 (2182.28)	138.35 (674.45)	743.06 (3499.84)	47.29 (18.48)	0.08 (0.07)
H (K)		12.300	18.219	4.734	3.530	0.972
*P* value		.006	*<*.001	.192	.317	.808
Lymph nodes						
Negative	56 (64.37%)	161.90 (345.05)	84.65 (165.88)	711.42 (552.86)	49.45 (7.45)	0.08 (0.03)
Positive	31 (35.63%)	714.50 (1074.40)	331.00 (859.80)	1332.02 (999.87)	44.45 (5.20)	0.12 (0.08)
Z value		−4.431	−4.768	−3.873	−3.036	−3.341
*P* value		*<*.001	*<*.001	*<*.001	.002	.001

Others: immature teratoma (one case), granulosa cell tumor (one case), endometrioid carcinoma (two cases), carcinosarcoma (two case).

FAR = fibrinogen(g/L)/ albumin(g/L), FIGO = International Federation of Gynecology and Obstetrics, IQR = interquartile range, OC = ovarian cancer, PNI = albumin (g/L) + 5 × lymphocyte count (10^9^/L), SII = platelet count (10^9^/L) × neutrophil count (10^9^/L)/ lymphocyte count (10^9^/L).

Next, we investigated the differences in variables with respect to age, histological grade, and pathological type. Table 2 shows no statistically significant differences in the variables between the different age groups (*P* = .968, *P* = .535, *P* = .685, *P* = .284, *P* = .704, respectively). However, CA125 and HE4 showed significant differences in categorical variables, such as histological grade (*P* = .049 and *P* = .002, respectively) and pathological type (*P* = .006 and *P* < .001, respectively). No statistically significant differences were found in SII, PNI, and FAR between histological grades (*P* = .292, *P* = .817, *P* = .429, respectively) and pathological types (*P* = .192, *P* = .317, *P* = .808, respectively).

### 3.3. Efficiency of single CA125, HE4, SII, PNI, FAR, and different combinations of the variables in the diagnosis of OC

**Figure 1.**
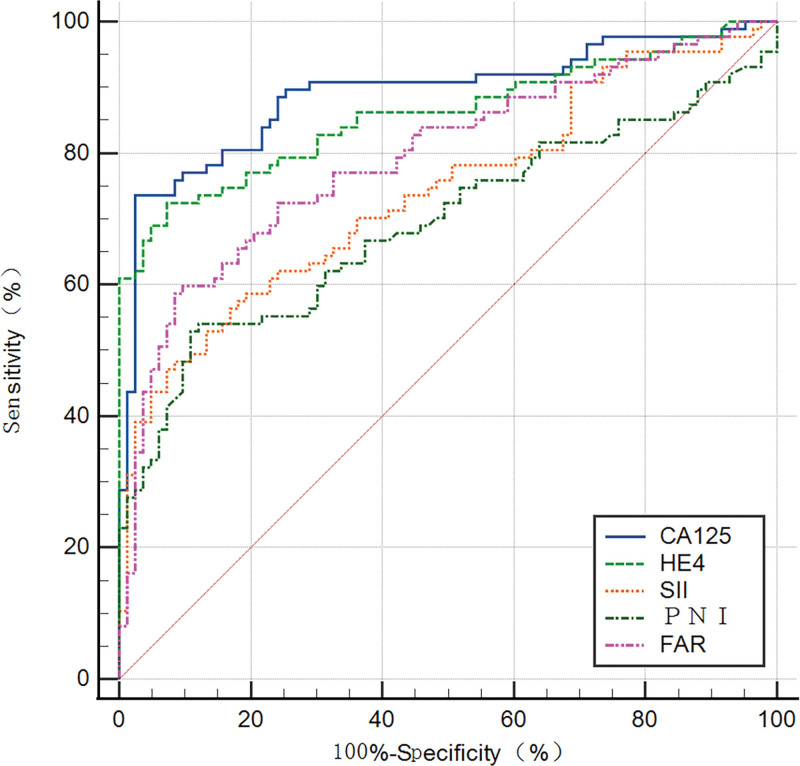
ROC curves of CA125, HE4, SII, PNI, and FAR in the diagnosis of OC. FAR = fibrinogen-to-albumin ratio, OC = ovarian cancer, PNI = prognostic nutritional index, ROC = operating characteristic curve, SII = systemic immune-inflammation index.

**Figure 2.**
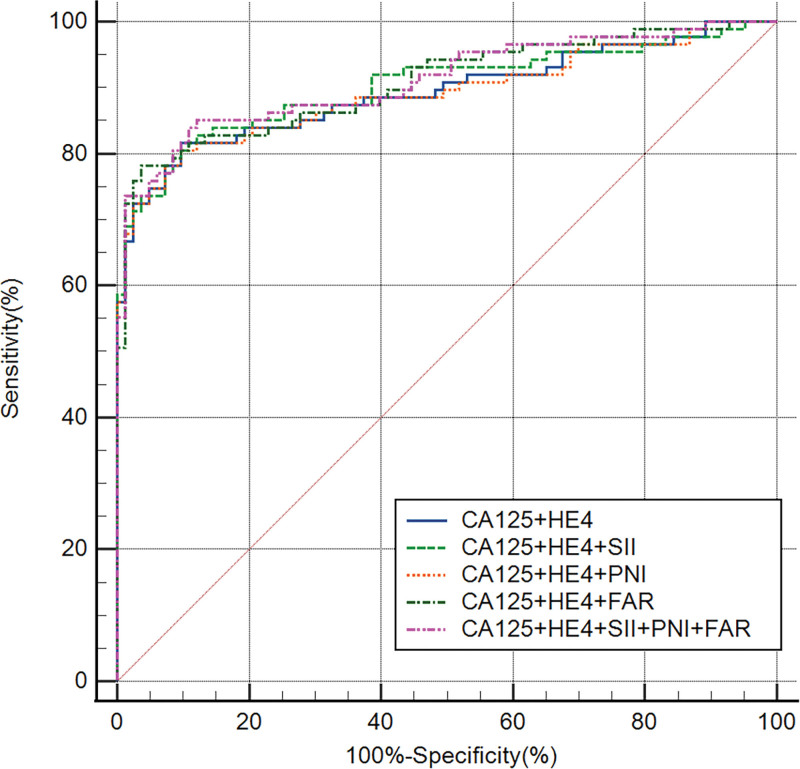
ROC curves of different combinations of variables in the diagnosis of OC. OC = ovarian cancer, ROC = operating characteristic curve.

**Figure 3.**
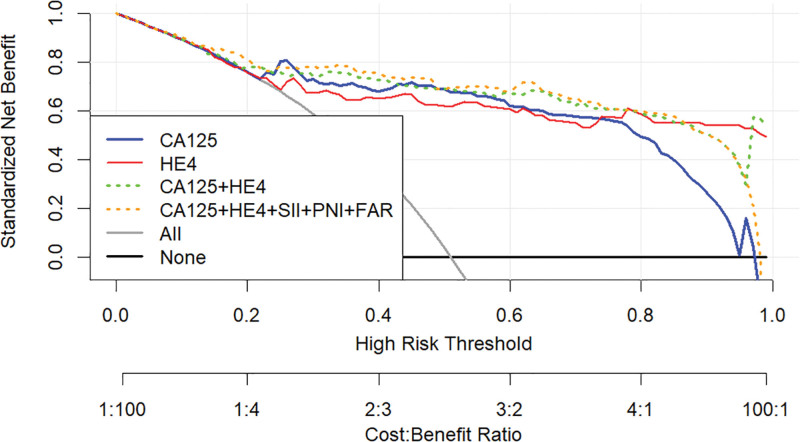
Decision curve showing the net benefit of CA125, HE4, CA125 + HE4, and CA125 + HE4 + SII + PNI + FAR in women at risk of developing ovarian cancer. CA125 dosage, positive if ≥ 79.89 U/mL, negative if < 79.89 U/mL; HE4 dosage, positive if ≥ 65.16 pmol/L, negative if < 65.16 pmol/L; FAR, positive if ≥ 0.084, negative if < 0.084; PNI, positive if ≥ 46.9, negative if < 46.9; SII, positive if ≥ 945.206, negative if < 945.206. FAR = fibrinogen-to-albumin ratio, PNI = prognostic nutritional index, SII = systemic immune-inflammation index.

Based on the association of CA125, HE4, SII, PNI, and FAR with OC, ROC curves were constructed and used to determine the optimal cutoff value and the corresponding sensitivity and specificity (Fig. 1). Table 3 presents the results. The optimum cutoff value was chosen to maximize the Youden index (sensitivity + specificity − 1). The appropriate cutoff value of CA125 [area under the ROC curve (AUC) = 0.890, *P<*.001], HE4 (AUC = 0.859, *P<*.001), FAR (AUC = 0.793, *P<*.001), SII (AUC = 0.739, *P<*.001), and PNI (AUC = 0.692, *P<*.001) for differentiating BOTs and OC were 79.89, 65.16, 0.084, 945.206, and 46.9, respectively; with the corresponding sensitivity of 73.6%, 72.4%, 58.6%, 47.1%, and 52.9%, respectively; specficity of 97.6%, 92.8%, 91.6%, 92.8%, and 89.2%, respectively; PPV of 95.52%, 90%, 88.14%, 85.42%, and 82.14%, respectively; NPV of 77.67%, 76%, 68.47%, 62.30%, and 62.39%, respectively; positive LR of 30.667, 10.056, 6.976, 6.542, and 4.898, respectively; negative LR of 0.270, 0.297, 0.452, 0.570, and 0.528, respectively; accuracy of 84.71, 81.76, 75.29, 68.82, and 70.0 respectively. The FAR tested showed the highest sensitivity (58.6%), PPV (88.14%), NPV (68.47%), positive LR (6.976), accuracy (75.29), and lowest negative LR (0.452) to differentiate BOTs from OC In the 3 inflammatory-nutritional indices (FAR, PNI, and SII). Table 3 also provides the relevant cutoff value, sensitivity, specificity, PPV, NPV, positive LR, negative LR, and accuracy of the various combinations of the defined variables for differentiating BOTs from OC. Overall, the CA125 tested, when compared separately, displayed the highest sensitivity (73.6%), specificity (97.6%), PPV (95.52%), NPV (77.67%), positive LR (30.667), accuracy (84.71%), and lowest negative LR (0.270) when it was used to differentiate BOTs from OC. The AUC of the combination of the 5 variables was higher than that of any other combination of the variables (Fig. 2). Compared with CA125 alone, the combination of the 5 variables showed a slight increase in sensitivity (83.91%), NPV (83.91%), and accuracy (85.88%) and a decrease in negative LR (0.180%) (Table 3). The DCA for CA125, HE4, and CA125 combined with HE4, as well as the combination of CA125, HE4, FAR, SII, and PNI are shown in Figure 3. This graphic analysis showed that the combination of CA125, HE4, FAR, SII, and PNI had higher clinical utility than isolated CA125 or HE4 or the combination of CA125 and HE4. When we combined CA125, HE4, FAR, SII, and PNI, we observed an enhancement of this clinical value, which was higher than the combination of CA125 and HE4 clinical values in the range of 15% to 75% risk thresholds.

**Table 3:** Cutoff value and diagnostic value of CA125, HE4, SII, PNI, FAR, and different combinations of the variables in the diagnosis of OC.

Variables	AUC	Cutoff	95% CI	*P* value	Sensitivity (%)	Specificity (%)	PPV (%)	NPV (%)	Positive LR	Negative LR	Accuracy (%)
CA125	0.89	79.89	0.833–0.933	*<*.001	73.6	97.6	95.52	77.67	30.667	0.270	84.71
HE4	0.859	65.16	0.797–0.908	*<*.001	72.4	92.8	90	76	10.056	0.297	81.76
FAR	0.793	0.084	0.724–0.851	*<*.001	58.6	91.6	88.14	68.47	6.976	0.452	75.29
SII	0.739	945.206	0.666–0.803	*<*.001	47.1	92.8	85.42	62.30	6.542	0.570	68.82
PNI	0.692	46.9	0.617–0.761	*<*.001	52.9	89.2	82.14	62.39	4.898	0.528	70.0
CA125 + HE4	0.893	0.338	0.836–0.935	*<*.001	81.6	90.36	88.75	82.22	8.465	0.204	85.29
CA125 + HE4 + SII	0.902	0.400	0.847–0.942	*<*.001	79.31	91.57	90.91	81.72	9.408	0.226	85.88
CA125 + HE4 + PNI	0.891	0.414	0.834–0.934	*<*.001	78.16	92.77	90.67	80.00	10.811	0.235	84.71
CA125 + HE4 + FAR	0.906	0.531	0.852–0.946	*<*.001	78.16	96.39	94.44	80.61	21.651	0.227	86.47
CA125 + HE4 + SII + PNI + FAR	0.910	0.350	0.857–0.949	*<*.001	83.91	89.16	87.95	83.91	7.74	0.180	85.88

AUC = area under the curve, CI = confidence interval, FAR = fibrinogen (g/L)/albumin (g/L), LR = likelihood ratio, NPV = negative predictive value, OC = ovarian cancer, PNI = albumin (g/L) + 5 × lymphocyte count (10^9^/L), PPV = positive predictive value, SII = platelet count (10^9^/L) × neutrophil count (10^9^/L)/lymphocyte count (10^9^/L).

Overall, compared to the early-stage OC (Stage I–II) group, CA125, HE4, SII, and FAR values in the advanced OC (Stage III–IV) group were significantly higher, while PNI was significantly lower. Isolated CA125 showed the best application value to differentiate BOTs from OC when the defined variables were compared separately. The combination of these 5 variables displayed a greater AUC than any one of them alone. Compared to CA125 alone, the combination of CA125, HE4, FAR, SII, and PNI showed a slight gain in sensitivity (83.91%), NPV (83.91%), and accuracy (85.88%) and a decrease in negative LR (0.180%). The 5 variables combined yielded a more advantageous clinical outcome than CA125 alone, HE4 alone, or combined CA125 and HE4. Higher preoperative CA125, HE4, SII, and FAR levels and lower PNI levels indicate a greater likelihood of advanced OC progression and lymph node metastasis. FAR has better application value than other inflammation-related markers (PNI and SII).

## 4. Discussion

The incidence of OC, the deadliest gynecological malignancy and a major contributor to cancer-related fatalities in women globally,^[25]^ has increased in recent years.^[2]^ <30% of patients survive because there are no early signs of OC or early screening or diagnosis.^[26]^ Given the low prognosis of this cancer, it is necessary to improve the survival rates of patients by using methods to accurately predict the risk factors that affect the severity of cancer and early diagnosis.

CA125 was first described in the early 1980s.^[27]^ In OC patients, serum CA125 levels may be higher. However, in stage I, only 23% to 50% of cases demonstrate sensitivity to this measurement; it is not particularly sensitive during the early stages of the disease.^[11]^ The specificity of CA125 for identifying OC was 78% (95% confidence interval 76–80) in a meta-analysis by Ferraro et al^[28]^ The AUC for CA125 in the study by Dikmen et al^[29]^ was relatively low (0.78), indicating that it was probably not an ideal marker for OC diagnosis.

In an effort to better detect OC in its early stages, new biological markers, such as HE4^[11]^ have been studied. Reports suggest that HE4 is overexpressed in ovarian tumors, particularly endometrioid OC.^[12]^ Moreover, HE4 is not highly expressed in clear-cell ovarian carcinomas as in other epithelial ovarian cancers.^[30]^ According to Yanaranop et al,^[31]^ HE4 had 86% specificity and the AUC was superior to that of CA125 alone, with values of 0.893 and 0.865, respectively.^[32]^ A recent Italian multi-center study suggested that HE4 may have at least partially different roles in EOC diagnosis than CA125.^[33]^ Moreover, HE4 was found to be more effective than CA125 in ruling EOC patients in both the disease and early stages of tumors.^[33]^ However, the CA125 test performed better than the HE4 test in terms of sensitivity (73.6%), specificity (97.6%), PPV (95.52%), NPV (77.67%), positive LR (30.667), accuracy (84.71%), and lower negative LR (0.270).

The immune response and systemic inflammatory processes have been revealed to be essential in the initiation and progression of various tumors.^[17]^ The immune system and inflammatory responses are linked to various stages of carcinogenesis, including initiation, invasion, promotion, and metastasis.^[34]^ Studies have shown that malnutrition can increase the likelihood of postoperative complications, increase the vulnerability of patients to infection, and encourage tumor recurrence by suppressing tumor immunity.^[18,19,35]^ The immune and nutritional states of the body are critical elements of inflammatory reactions. Cancer malnutrition typically results from the activation of systemic inflammation caused by disease progression, which impairs immunity and decreases survival.^[19,36,37]^ In addition, patients with advanced OC often suffer from malnutrition due to peritoneal dissemination caused by intestinal obstruction.^[38]^ The role of inflammation and nutrition in cancer patients has been the focus of a growing number of studies. Inflammation is a major factor in the tumor microenvironment.^[39]^ Many inflammatory cells and cytokines in the tumor microenvironment can affect the growth, development, and metastasis of cancer.

Platelets, neutrophils, and lymphocytes, which can aggregate in vessels and release factors, such as vascular endothelial growth factor, TGF-β, and platelet-derived growth factor, can affect the biological behavior of cancer cells.^[40–44]^ Thrombopoietin and inflammatory mediators released by cancer cells may stimulate platelet growth, and in turn, tumor growth. Neutrophils, by releasing VEGF and matrix metalloproteinase, can promote angiogenesis, tumor growth, and metastasis.^[39]^ Lymphocytes are responsible for the immune defense against tumor cells by releasing tumor necrosis factor, interferon-γ, and other cytokines. When their levels are low, these cytokines may create a favorable tumor microenvironment for cancer cells to proliferate, progress, and spread. At the same time, the body’s immune system may be weakened by a reduction in the number of lymphocytes, and cancer cells are more likely to escape the immune system, leading to poor prognosis for cancer patients. Ostroumov et al^[45]^ reported that CD4 and CD8 T lymphocytes can mediate cancer cell growth.

The SII, which accounts for the peripheral blood counts of platelets, neutrophils, and lymphocytes [SII = (P*N)/L], can represent different inflammatory and immune pathways in the body and has greater stability.^[46]^ It has been used in the diagnosis and treatment of a range of malignant tumors and is associated with patient prognosis, indicating the immunological and inflammatory status of patients with malignant tumors.^[47–49]^ This marker has greater predictive power than either the neutrophil-to-lymphocyte ratio or the platelet-to-lymphocyte ratio^[50–52]^ and has been linked to a decrease in both overall survival (OS) and disease-free survival rates in patients with colon cancer, OC, or hepatocarcinoma. The SII has been evaluated as a relevant prognostic factor for OC, but few studies have focused on its role in preoperatively predicting malignancy.

Albumin is a significant indicator of acute-phase proteins and systemic chronic inflammation.^[53]^ Its use is widespread in reflecting the overall nutritional status of the body and is considered a promising prognostic factor for various malignancies.^[54,55]^ During inflammation, pro-inflammatory cytokines such as interleukin-1, interleukin-6, and tumor necrosis factor-α can inhibit the production of albumin.^[56,57]^ A low serum albumin level may indicate that the host is experiencing malnutrition, which can negatively impact the overall health. This state of malnourishment can impair the body’s defense mechanisms, such as cellular immunity, humoral immunity, and phagocytic functioning.^[58]^ A meta-analysis demonstrated that preoperative serum albumin levels could be employed as an independent prognostic indicator of OS in patients with EOC.^[59]^ It has been well demonstrated that low serum albumin concentrations are associated with poor survival in EOC patients.

PNI and SII are known to reflect systemic inflammatory status. PNI, calculated by the serum albumin concentration and peripheral blood lymphocyte count, can reflect both the nutritional and immunological status of the host and has been validated as an indicator for predicting short- and long-term prognoses.^[60]^ Recently, it was discovered that a low PNI was linked to a poor prognosis for HGSOC and cervical cancer.^[61,62]^ The prognostic value of pretreatment PNI has been verified in several tumors such as pancreatic cancer,^[63]^ liver cancer,^[64]^ and colorectal cancer.^[65]^ Mounting evidence suggests that the preoperative PNI could serve as an indicator of prognosis for patients with OC.^[60,66,67]^ According to Miao et al^[68]^, PNI is an independent prognostic indicator of OS and progression-free survival in patients with OC. However, it has rarely been reported as an indicator for OC diagnosis.

Plasma fibrinogen is another acute-phase protein characterized by elevated levels during systemic inflammation.^[69]^ It is considered a key factor in the regulation of inflammation and cancer development by mediating the proliferation and migration of tumor cells, as well as the initiation of angiogenesis.^[70]^ The potential of fibrinogen as a molecular bridge between angiogenesis and tumor cell growth is of great significance,^[71]^ as it can also impede natural killer cells from attacking tumor cells or cytotoxic agents that target them, thus stimulating tumor cell growth and allowing them to evade immune surveillance. This mechanism promotes tumor cell growth.^[72]^ Various studies have consistently revealed that elevated fibrinogen levels prior to treatment are associated with unfavorable prognosis in various cancers.^[73,74]^ A study conducted by Perisanidis et al^[75]^ in 2015 demonstrated that fibrinogen can serve as an independent prognostic biomarker in patients with cancer. It has also been found to potentially contribute to tumor growth and metastasis potentially.^[76]^

The FAR, which considers both fibrinogen and albumin, has recently been identified as a prognostic factor for various malignancies.^[77–82]^ One potential approach to improve the accuracy of assessing inflammation and nutritional status in patients is to combine fibrinogen and albumin levels with FAR. Elevated serum fibrinogen and decreased serum albumin levels are widely recognized as effective biomarkers for detecting elevated systemic inflammation.^[75,83]^ According to research conducted by Xie et al^[84]^, a high FAR could indicate more aggressive tumor biological characteristics as well as progressive systemic inflammation. In addition, FAR could amplify the sensitivity of inflammation and nutritional status in patients with EOC, and it was found to be more effective than fibrinogen or albumin alone in predicting the prognosis of EOC.

Moreover, based on the ROC curve analysis, FAR was found to have superior utility compared to other inflammation-related markers such as neutrophil-to-lymphocyte ratio, platelet-to-lymphocyte ratio, and PNI. Notably, although both FAR and PNI incorporated albumin, FAR had a more pronounced impact on prognosis, possibly because of its greater emphasis on fibrinogen levels. However, there are no studies on the use of FAR in OC diagnosis, and the role of FAR in OC remains relatively unexplored. In this study, the FAR test showed the highest sensitivity (58.6%), PPV (88.14%), NPV (68.47%), positive LR (6.976), accuracy (75.29), and lowest negative LR (0.452) to differentiate BOTs from OC among the 3 inflammatory-nutritional indices (FAR, PNI, SII).

The present study included 170 patients with documented OC and BOT. This demonstrated that preoperative CA125 combined with HE4, SII, PNI, and FAR compensated for single-use shortcomings, ensuring a high sensitivity, NPV, and accuracy. To the best of our knowledge, no study has been published up to now that examined the clinical utility of CA125 combined with HE4, SII, PNI, and FAR in predicting OC in the preoperative setting. Our results suggest that the preoperative serum SII, PNI, and FAR may be clinically valuable markers in patients with OC. Of the 3 inflammatory-nutritional indices tested (FAR, PNI, and SII), FAR showed the highest sensitivity (58.6%), PPV (88.14%), NPV (68.47%), positive LR (6.976), accuracy (75.29), and lowest negative LR (0.452) for differentiating between BOTs and OC. The combination of CA125, HE4, SII, PNI, and FAR had better application values than other combinations of the 5 variables. Compared with the early-stage OC (Stage I–II) group, CA125, HE4, SII, and FAR values in the advanced OC (Stage III–IV) group were significantly higher. In contrast, the PNI value in the advanced OC (stages III–IV) group was significantly lower. Based on the results of previous studies and the current study, it is believed that FAR and SII increase and PNI decreases as cancer progresses. Consequently, an increase in the FAR and SII and a decrease in the PNI are linked to advanced ovarian cancer and could indicate a poor prognosis. FAR has better application value than other inflammation-related markers (PNI and SII). In the present study, DCA showed that the combination of CA125, HE4, FAR, SII, and PNI had a higher net benefit than CA125, HE4, or CA125 combined with HE4. The inflammation-related marker changes were not cancer-specific. An increase or decrease in these values does not indicate an absolute risk of ovarian cancer. However, inflammation-related markers with differential counts are common and inexpensive preoperative markers. Therefore, even if it is not a confirmatory test for OC, it has clinical utility as a potential auxiliary tool for preoperative differential diagnoses. Our study showed that the addition of inflammatory biomarkers to CA125 and HE4 can achieve satisfactory efficiency in distinguishing BOTs from OC. Preoperative biomarkers provide some assistance. However, the management process often relies on imaging examinations once abnormal serum biomarker results are detected. As we delve deeper into the inflammatory mechanisms associated with tumors, we may discover more effective combinations of tumor and inflammatory biomarkers.

It is important to note that this retrospective study included a relatively small number of patients from a single center. However, the rigorous inclusion and exclusion criteria and high statistical significance achieved for the diagnostic traits tested in our study provided strong evidence for the reliability and reproducibility of our findings.

## 5. Conclusion

This study suggests that preoperative serum SII, PNI, and FAR may be clinically valuable markers in patients with OC. The combination of CA125, HE4, SII, PNI, and FAR had better application values than other combinations of the 5 variables. It was superior to CA125, HE4, SII, PNI, and FAR levels alone in predicting OC in the preoperative setting. Higher preoperative CA125, HE4, SII, and FAR levels, and lower PNI levels predicted a higher probability of advanced OC progression and lymph node metastasis. FAR has better application value than other inflammation-related markers (PNI and SII). As we delve deeper into the inflammatory mechanisms associated with tumors, we may discover more effective combinations of tumor and inflammatory biomarkers. The main limitation of our study is that it was a retrospective analysis with a small sample of patients from a single center and the same region. Thus, a broader multi-center study to validate the results and attain a higher statistical power would be interesting.

## Acknowledgments

We would like to thank all doctors, nurses, patients, and their family members for their kindness in supporting this study.

## Author contributions

**Conceptualization:** Liyun Song, Jie Qi, Jing Zhao, Ren Xu.

**Data curation:** Liyun Song, Jie Qi, Suning Bai, Ren Xu.

**Formal analysis:** Liyun Song, Jie Qi, Jing Zhao.

**Investigation:** Liyun Song.

**Methodology:** Qi Wu.

**Software:** Liyun Song, Jie Qi, Jing Zhao, Suning Bai, Qi Wu, Ren Xu.

**Supervision:** Liyun Song.

**Writing – original draft:** Liyun Song, Jie Qi, Jing Zhao, Suning Bai, Qi Wu.

**Writing – review & editing:** Liyun Song.
